# The influence of cerebrospinal fluid on epidermal neural crest stem cells may pave the path for cell-based therapy

**DOI:** 10.1186/scrt235

**Published:** 2013-07-18

**Authors:** Sareh Pandamooz, Mohammad Naji, Farid Alinezhad, Amin Zarghami, Mohsen Pourghasem

**Affiliations:** 1 Cellular and Molecular Biology Research Center, Babol University of Medical Sciences, Babol, Iran; 2 Department of Biology, Kharazmi University, Tehran, Iran; 3 Department of Anatomy, School of medicine, Tehran University of Medical Sciences, Tehran, Iran; 4 Student Research Committee, Babol University of Medical Sciences, Babol, Iran

**Keywords:** Epidermal neural crest stem cell, Cerebrospinal fluid, Hair follicle, Bulge

## Abstract

**Introduction:**

Epidermal neural crest stem cells (EPI-NCSCs) in the bulge of hair follicles are a promising source for cell-replacement therapies in neurodegenerative diseases. A prominent factor in cell-based therapy is the practicalities of different routes of administration. Cerebrospinal fluid (CSF), owing to its adaptive library of secreted growth factors, can provide a trophic environment for transplanted cells. Thus, the effect of CSF on the behavior of EPI-NCSC was studied here.

**Methods:**

In this study, the highly pure population of EPI-NCSCs was obtained from the bulge of mouse hair follicle. Migrated cells were characterized with real-time polymerase chain reaction (RT-PCR) and immunocytochemistry. Subsequently isolated stem cells were cultured in CSF, which was collected from the cisterna magna of the adult rat. The expression of pertinent markers was assessed at the gene and protein levels with RT-PCR and immunocytochemistry, respectively. Colorimetric immunoassay was used to quantify the rate of proliferation of EPI-NCSCs after cultivation in CSF.

**Results:**

Isolated EPI-NCSCs could survive in the CSF, and they maintained the expression of nestin, β–tubulin ІІІ (early neuronal marker), and glial fibrillary acidic protein (GFAP, glia marker) in this environment. In addition, CSF decreased the proliferation rate of EPI-NCSCs significantly in comparison to primary and expansion culture medium.

**Conclusions:**

Our findings demonstrate that CSF as a cocktail of growth factors helps EPI-NCSCs to acquire some desirable traits, and because of its circulatory system that is in close contact with different parts of the central nervous system (CNS), can be a practical route of administration for delivery of injected stem cells.

## Introduction

Epidermal neural crest stem cells (EPI-NCSCs) are multipotent stem cells that persist in the bulge of hair follicles through adulthood, and they have the capacity to generate various types of differentiated cells under appropriate culture conditions [1]. EPI-NCSCs are one of the main derivatives of transient embryonic neural crest that retain the neurologic differentiation potential of their neural crest origin. The neural crest forms during gastrulation and locates in the boundary between somatic ectoderm and neuroectoderm. It leaves the closing neural tube during late neurulation, invades the embryo, and give rise to distinct cell types and tissues, such as craniofacial bone/cartilage, meninges, tooth papillae, the autonomic and enteric nervous systems, sensory ganglia, endocrine cells of the adrenal medulla, smooth musculature of the cardiac outflow tract, and great vessels and pigment cells (melanocytes) of the skin and internal organs. The bulge region within the outer root sheath of the hair follicle is one of the prime targets of the neural crest during development that serves as a specialized niche for epidermal stem cells [2-6]. EPI-NCSCs exhibit several characteristics of embryonic and adult stem cells. Similar to embryonic stem cells, these cells show a high level of physiological plasticity, and they can be easily expanded under *in vitro* condition. Similar to other kinds of adult stem cells, they are a promising group of stem cells that do not elevate ethical concern. Despite all these similarities, this unique type of stem cells can circumvent several setbacks associated with embryonic stem cells, such as immunologic incompatibility. Moreover, they are relatively abundant and accessible in the bulge area of hairy skin and can be isolated by a minimally invasive procedure. However, most of other types of adult stem cells are fairly sparse and approachable with difficulty [7-9].

Previous studies have established that local signaling and regional identity during migration of neural crest cells play a crucial role in cell-type specification, and several investigations have emphasized on the importance of the concerted action of a combination of growth factors on survival, proliferation, and differentiation of neural crest cells at multiple levels [10,11]. Therefore, it is quite conceivable that the CSF, as a cocktail of secreted growth factors, can provide a trophic environment for survival and proliferation of these multipotent stem cells. This issue has received support from numerous studies that examined the critical influence of CSF-borne signals not only on neuroectodermal cells during brain development but also on survival, proliferation, and fate specification of neural stem cells in adult brain throughout life [12-18]. Furthermore, the close ontologic relation between EPI-NCSCs and stem cells of the central nervous system (CNS) has fueled this hypothesis that the CSF can be an instructive milieu for these cells because the fate of neural progenitor cells at the brain-CSF interface is governed by CSF [19,20].

Based on these facts, in this experiment, the influence of CSF on the EPI-NCSCs was studied to demonstrate whether it can help these cells to acquire some desirable traits that establish them as an appealing candidate for cell-replacement therapy in different CNS injuries and neurodegenerative diseases.

## Materials and methods

All experimental protocols of this study were approved by local ethics committee at Babol University of medical sciences.

### Cerebrospinal fluid collection

CSF was collected from the cisterna magna (CM) of Wistar rats with 200 to 300 g of body weight by using a fire-polished 1-ml syringe connected to a 27G dental needle. Here the animal was anesthetized with xylazine 2% and ketamine 50 mg/kg per body weight intraperitoneally and placed on the stereotaxic instrument (Stoelting, Wood Dale, IL, USA). Specially constructed ear bars were placed in the external auditory meatus, and the head was flexed downward at approximately 90 degrees so that the occipital bone was almost horizontal. A median incision was made, and the cervicospinal muscle was reflected and the posterior atlanto-occipital membrane exposed.The needle was inserted vertically and centrally to the depressible surface with a rhomboid appearance between the occipital protuberance and the spine of the atlas. A gentle aspiration flow the CSF through the syringe. Collected CSF was transferred to a sterile microtube on ice and centrifuged (Sigma, Osterode am Harz, Germany) at 10,000 rpm for 10 minutes to remove cells or debris, and ultimately all supernatants were stored at −80°C until use. Because the volume of collected CSF from each rat was approximately 100 μl, to provide adequate volume of CSF for the experiment, it all was pooled.

### Chick embryo extract preparation

The head of the day-11 chick embryo was cut off. Then the embryo was chopped, homogenized with an equal volume of HBSS (PAA, Austria), and the mixture was incubated for 30 minutes on ice. Subsequently, it was centrifuged at 12,000 rpm for 30 minutes at 4°C, and the supernatant was removed and passed through 0.45-μm and 0.22-μm filters sequentially.

### Dissection of the bulge from adult whisker follicle

**Figure 1:**
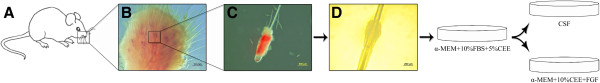
**Schematic view of EPI-NCSC isolation and culture.** The whisker pad **(B)** of 3-week-old mouse **(A)** was cut, and hair follicles **(C)** were dissected. The bulge region **(D)** was rolled out from the capsule of hair follicle and explanted in α-MEM, 10% FBS, and 5% CEE (primary explant). After 10 days, migrated EPI-NCSCs were trypsinized and cultured in α-MEM supplemented with 10% CEE and FGF (expansion medium) or cells cultivated in only CSF for 72 hours.

The bulges of hair follicles were microdissected from whiskers of 3 week-old NMRI mice as described previously [1]. In brief, pups were killed by cervical dislocation, and follicles of the whisker pad were dissected, cleaned, and cut longitudinally and then transversely (below and above the bulge region). Subsequently, the bulges were rolled out of their capsules and explanted into collagen-coated 24-well culture plates (Roche, Mannheim, Germany; TPP, Switzerland) (Figure 1).

### Isolation and *in - vitro* expansion of EPI-NCSCs

Explanted bulges were cultured in Alpha-modified MEM (PAA, Pasching, Austria) supplemented with 10% fetal bovine serum (FBS) (PAA), 5% day-11 chick embryo extract (CEE), and 1% penicillin/streptomycin (PAA). Fifty percent of the culture medium was exchanged every other day. After observation of migrated cells, the bulge explants were carefully removed with a 27G needle to minimize the rate of contamination with other undesirable later-migrating cell types, such as keratinocytes. Adhering EPI-NCSCs were resuspended by trypsinization, placed in fresh collagen-coated plates at 7 × 10^4^ cells per each well of four-well plate (SPL Life Sciences, Pochun, South Korea), and cultured for another 24 hours. Thereafter, isolated EPI-NCSCs were cultured in two different mediums. The first group of cells was expanded in the culture medium that consisted of 90% Alpha-modified MEM plus 10% day-11 chick embryo extract supplemented with fibroblast growth factor-2 (FGF-2, 20 ng/ml; Sigma-Aldrich, St. Louis, MO, USA). Likewise, a second group of cells were cultivated in the collected CSF for 72 hours.

### Immunofluorescent staining

Indirect immunofluorescent staining was performed to demonstrate the presence of Nestin, β-tubulin ІІІ and GFAP-positive cells in the population of migrated cells or cells after their cultivation in different media. Cultures were washed twice with phosphate-buffered saline (PBS; PAA) for 2 minutes and fixed with 4% paraformaldehyde (Merck, Germany) in PBS at room temperature (RT) for 12 minutes, followed by three 5-minute TPBS washes (0.05% Tween-20 in PBS, Sigma-Aldrich). They were permeabilized with 0.2% Triton X-100 (Merck) in PBS at RT for 10 minutes and then were washed again in TPBS for three 5–minute intervals, and blocked with 1% BSA in TPBS at RT for 1 hour. Primary antibody was added, diluted appropriately in blocking buffer, and incubated overnight at 4°C. Cultures were then rinsed 3 times with TPBS for 5 minutes each, followed by addition of secondary conjugated antibody diluted (1:250) in blocking buffer and incubated in the dark for 1 hour at RT. Secondary antibody was removed, and three, 5-minute TPBS washes were performed, followed by counterstaining with propidium iodide (PI) (Sigma-Aldrich). Primary antibodies used included rabbit anti-Nestin (1:200) (Abcam, Cambridge, UK.), rabbit anti-β-tubulin ІІІ (1:50) (Sigma-Aldrich), and rabbit anti-GFAP (1:1,000) (Abcam). Goat anti-rabbit IgG was used as secondary antibody (Sigma-Aldrich).

### RNA extraction, cDNA synthesis and polymerase chain reaction

Total RNA was extracted by using RNX-Plus solution (Cinnagen, Iran) followed by genomic DNA digestion with RNase-free DNase I (Thermo Scientific, Waltham, MA, USA). RNA concentration was quantified by WPA spectrophotometer (Biochrom) and Hellma lens (Hellma Analytics). Subsequently 500 ng of DNA-free RNA was reverse transcribed by using the RevertAid first-strand cDNA synthesis kit per manufacturer’s instructions (Thermo Scientific). Primers for reverse transcription PCR were designed by AlleleID 6 software on exon junctions or spanned long introns. Primer sequence and amplicon lengths are listed in Table 1. Hypoxanthine guanine phosphoribosyl transferase (*Hprt*) served as a housekeeping gene for normalization. Conventional PCR was carried out to verify the EPI-NCSC origin of isolated cells. In brief, for each 25-μl PCR reaction, these components were mixed: 2.5 μl PCR buffer, 0.5 μl dNTP mix, 0.75 μl of 50 m*M* MgCl_2,_ 0.5 μl of each primer, 1 μl cDNA template, 0.625 unit Taq enzyme, and the required volume of distilled water. Thermocycling parameters were 94°C for 3 minutes and 35 cycles of 94°C for 30 seconds, 60°C for 30 seconds, and 72°C for 1 minute by using Mastercycler gradient system (Eppendorf, Germany). To inspect PCR products, 5 μl of each PCR product was loaded onto 2% agarose gel and stained with GelRed (Biotium, Hayward, CA, USA) dye for 30 minutes. Gels were visualized with UV illumination, and images were captured by using GENE FASH gel-documentation system (Syngene Bio Imaging).

**Table 1:** Real-time PCR primers

**Gene**	**Forward primer (5′-3′)**	**Reverse primer (5′-3′)**	**Amplicon length**
***Sox-10***	TAGCCGACCAGTACCCTCAC	GCCTCTCAGCCTCCTCAATG	114
***Nestin***	AAGCAGGGTCTACAGAGTCAG	AGTTCTCAGCCTCCAGCAG	121
***GFAP***	GAGAAAGGTTGAATCGCTGGAG	GCTGTGAGGTCTGGCTTGG	138
***β – Tubulin ІІІ***	CCGCCTGCCTTTTCGTCTC	GGTCTATGCCGTGCTCATCG	131
***Hprt***	GGGCTTACCTCACTGCTTTC	CTGGTTCATCATCGCTAATCAC	137

For quantitative comparison, compatibility of each primer set efficiency with Hprt primers was validated by qRT-PCR of dilution series of cDNA templates. Each reaction (20 μl) consisted of 10 μl 2× SYBR Premix Ex TaqII (Takara), 0.8 μl forward primer, 0.8 μl reverse primer, 2 μl first-strand cDNA template (1:3 in distilled water), and 6.4 μl distilled water. Thermocycling was conducted as follows: 95°C for 30 seconds to activate HotStart enzyme, 40 cycles of 95°C for 5 seconds followed by 60°C for 35 seconds by using a Rotor-Gene Q instrument (Qiagen). At the end of each run, melting-curve analysis was performed, and a single amplification peak was considered specific amplification. Ct values of target genes were normalized against Hprt Ct (∆Ct), and the relative expression of each target was determined by using the ∆∆Ct method.

### Colorimetric immunoassay of cell proliferation

To quantify the proliferation of EPI-NCSCs in different medium, after trypsinization and counting, they were seeded at a density of 2.5 × 10^4^ cells/μl in a 96-well plate (Orange Scientific, Belgium) and after 24 hours, they were cultivated in four different mediums, (a) α-MEM, (b) α-MEM with 10% FBS and 5% CEE, (c) α-MEM supplemented with 5% CEE and FGF, and (d) 100% CSF. Cultures were maintained at 37°C in a 5% CO_2_ atmosphere for a further 72 hours. Cell proliferation was determined by using the colorimetric BrdU enzyme-linked immunosorbent assay (ELISA) kit (Roche, Mannheim, Germany), based on incorporation of BrdU during DNA synthesis in proliferating cells. Measurement was made on an ELISA reader (Rayto, China) at 450 nm.

### Imaging and statistical analysis

Images were obtained with the Olympus Stereomicroscope (SZX16) and invert florescence microscope (CKX41). Statistical analyses were performed on version 18 of SPSS statistical software (SPSS Inc. Chicago, IL, USA) and GraphPad Prism (Version 6.02, 1992–2013 GraphPad Software, Inc.) by using one-way ANOVA and the Tukey *post hoc* test. A value of *P* < 0.05 was considered significant.

## Results

### Expression of nestin, SOX10, β-tubulin ІІІ, and GFAP in culture of primary explants

**Figure 2:**
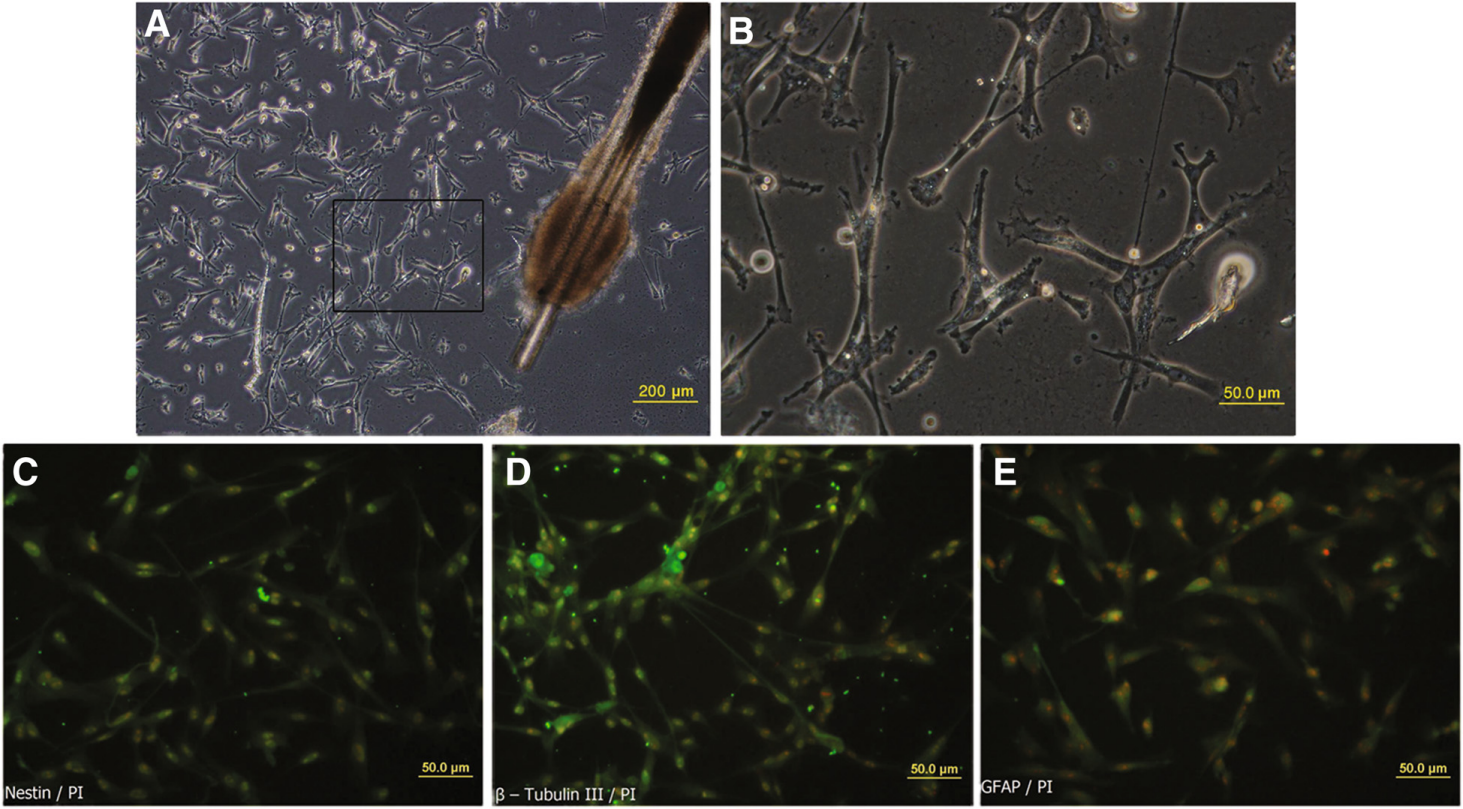
**Characteristics of epidermal neural crest stem cells. (A)** Morphology of cells 4 days after onset of EPI-NCSCs emigration from bulge explants. **(B)** Higher magnification of boxed area in **(A)**. **(C-E)** Expression of Nestin, β-tubulin ІІІ, and GFAP (all green) in the population of migrated cells 2 weeks after explantation .Nuclei are stained red with PI.

**Figure 3:**
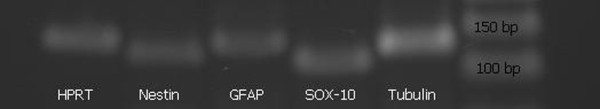
**Expression of key markers of EPI-NCSCs in primary explants.** RT-PCR analysis of expression of the neural crest stem cell genes (*SOX10* and *Nestin*) and early-lineage genes (*β-tubulin ІІІ* and *GFAP*) in primary explants. Expression of these genes validates the neural crest origin of migrated cells.

Within 2 to 3 days after explantation, cells with stellate morphology emigrated from whisker bulges with increasing numbers over time (Figure 2A, B). The phenotype of migrated cells from bulge explants was confirmed at gene and protein levels with RT-PCR and immunocytochemistry, respectively. After 10 days of cultivation of explanted bulges in culture medium containing α-MEM with 10% FBS and 5% CEE, the RT-PCR revealed the neural crest stem cell markers, SOX10, the progenitor cell marker, Nestin, glial marker, GFAP, and immature neurons marker, β-tubulin ІІІ; all were expressed in migrated cells of primary explants (Figure 3). After prolonged cultivation of migrated EPI-NCSC in primary media (2 weeks), cells spontaneously differentiate into neural crest progeny, which was confirmed by immunostaining of migrated cells from several explanted bulges in different wells with antibodies against Nestin, β-tubulin ІІІ, and GFAP separately (Figure 2C, E). Together, these observations validate expression of pertinent markers and characterize the bulge-derived cells as neural crest-derived cells.

### Expression of EPI-NCSC markers proceeds in CSF environment

**Figure 4:**
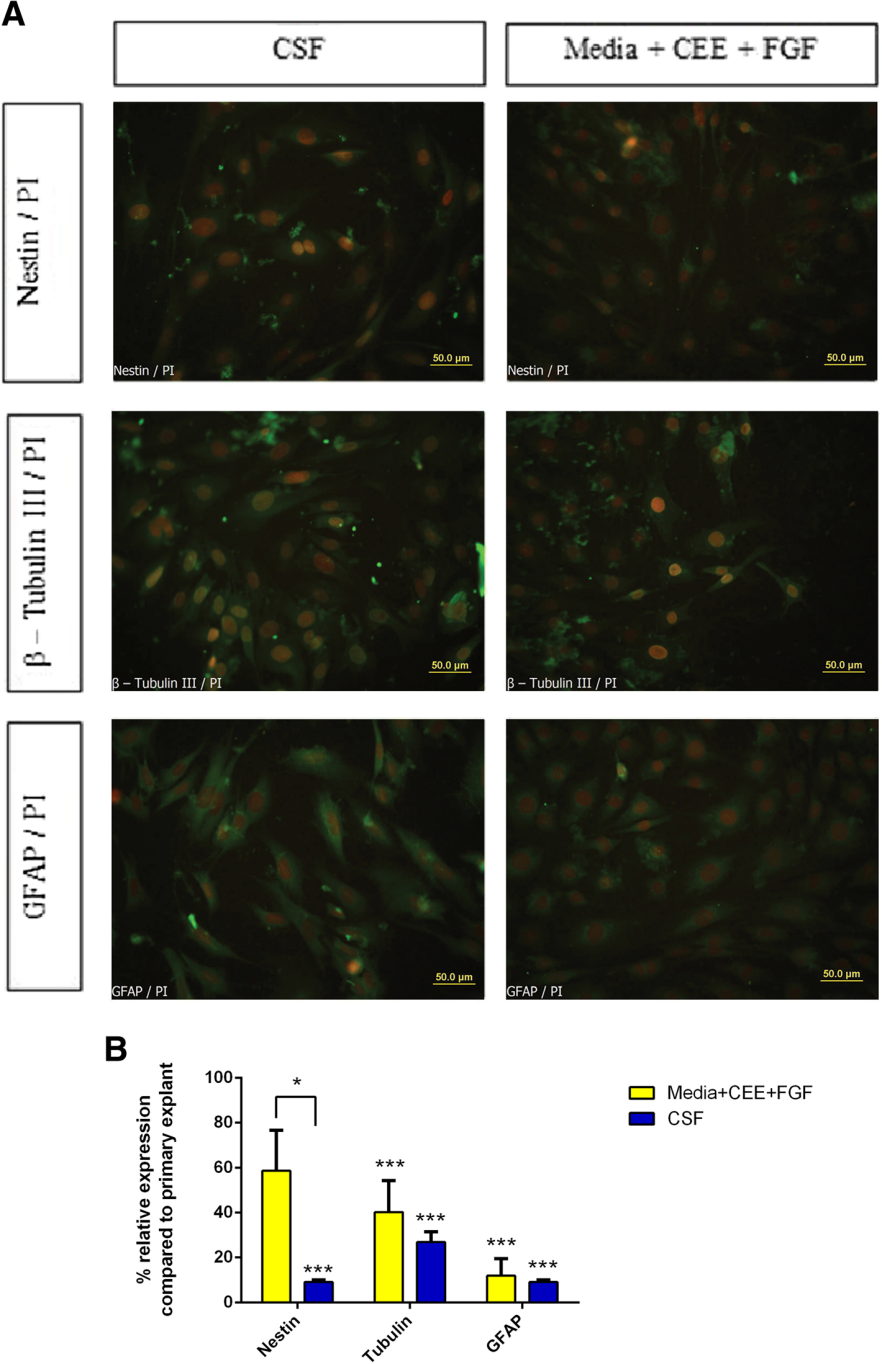
**Expression of pertinent genes of EPI-NCSCs. (A)** Immunocytochemistry of *Nestin, β-tubulin ІІІ*, and *GFAP* (all green) in expanded EPI-NCSCs after 72 hours of cultivation in CSF and expansion medium. All nuclei are stained red with PI. **(B)** Expression of *Nestin, β-tubulin ІІІ*, and *GFAP* genes in EPI-NCSCs was compared with primary explant by qRT-PCR, and the ΔΔ Ct method was used to determine alterations in expression levels after cultivation in expansion medium and CSF. qRT-PCR showed a decreased trend in expression of all three investigated genes after 72 hours of cultivation in CSF. *P < 0.05; ***P < 0.001. Error bars, 95% Cl.

The influence of CSF on characteristics of isolated EPI-NCSCs was investigated with immunocytochemistry and real-time PCR. Immunostaining of expanded EPI-NCSCs after 72 hours of cultivation in CSF indicated that these cells maintained the expression of *Nestin*, *β-tubulin ІІІ*, and *GFAP*, likewise cells that were plated in culture medium supplemented with 10% CEE and FGF. Close inspection of the cultures revealed that no cells with high expression of either GFAP or β-tubulin ІІІ markers were detected, supporting the notion that neither glia nor differentiated neurons were present in both groups of cells in two expansion media (Figure 4A). Also three established transcripts, Nestin, β-tubulin ІІІ, and GFAP were evaluated in EPI-NCSCs after cultivation in both CSF and expansion medium (α-MEM + CEE + FGF) by using qRT-PCR. Herein, the expression of mentioned genes was calibrated with the expression of those in the primary explants (α-MEM + FBS + CEE). The quantitative real-time PCR data showed the expression of Nestin and β-tubulin ІІІ and GFAP significantly decreased after 72 hours of cultivation in CSF (*P* < 0.001). Although the expression of nestin in CSF-cultivated cells was significantly lower than those in expansion medium (*P* < 0.05), expression of the other two genes is relatively unchanged between two groups of cells after cultivation in CSF and expansion medium, which indicates that these two culture conditions may share similarities in gene-expression patterns (Figure 4B).

### CSF decreases proliferation of isolated EPI-NCSCs

**Figure 5:**
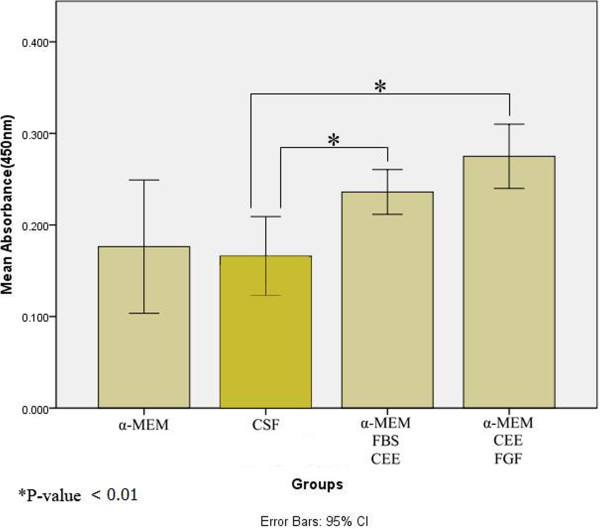
**Effect of CSF on the *in vitro *proliferation of isolated epidermal neural crest stem cells.** EPI-NCSCs were cultured in α-MEM only ( first group) or CSF (second group), or α-MEM with 10% FBS and 5% CEE (third group), or α-MEM supplemented with 10% CEE and FGF (fourth group) for 72 hours, and proliferation was assessed with a colorimetric BrdU ELISA kit. Proliferation of cells in either CSF or α-MEM was significantly lower than in cells in both supplemented α-MEMs. These data did not show significant differences between the rate of proliferation of cells in primary medium and expansion medium (third and fourth groups, respectively). ^*^P < 0.01. Error bars, 95% CI.

The influence of CSF on the self-renewal potential of isolated EPI-NCSCs was determined with a colorimetric BrdU ELISA kit after 72 hours of cultivation. The immunoassay indicated that the rate of proliferation was significantly reduced in CSF media compared with the primary culture and expansion medium (*P* = 0.009 and *P* = 0.001). This assay revealed that the proliferation of EPI-NCSCs in CSF is identical to that of cells that had been cultured in α-MEM (Figure 5). These data illustrate that CSF alone is able to function as a medium that maintain stem cells in a viable state and, because of its composition, support proliferation of cells at a lower rate.

## Discussion

A wide range of CNS injuries and neurodegenerative diseases results in various degree of cell death and neuroinflammation. Several therapeutic approaches have been evaluated for treatment of CNS impairment, and stem cell therapy is one of the promising means to achieve this aim. Cell-based therapies have recruited different types of stem cells to replace lost cells or to repair damaged areas. Studying the behavior of these cells after implantation and the feasibility of the mode of administration are two main debatable topics in cell-based therapies [21].

This investigation revealed that CSF, due to its beneficial environment, can retain viability of epidermal neural crest stem cells, and these cells continue to express a neural crest stem cell molecular signature after 72 hours of cultivation in the CSF milieu. According to our findings in the current study, CSF can be a suitable route of administration for EPI-NCSCs in CNS injuries and neurodegenerative diseases.

Previous studies have shown that EPI-NCSCs, as adult-resident stem cells in the bulge of the hair follicle, presents a number of advantages that make it an appropriate cell type for autologous transplantation. These readily accessible stem cells can generate several types of cells without known tumorigenic effects. Furthermore, their potential for regeneration of peripheral nerves and spinal cord injuries was demonstrated previously [22]. As neural crest stem cells are ontologically related to spinal cord stem cells, EPI-NCSCs are particularly attractive types of stem cell for treatment of spinal cord injury [23]. Several studies in mouse models of spinal cord injury showed that EPI-NCSC grafts resulted in significant improvement in sensory connectivity and touch perception. These cells modulate scar formation by contributing to the vascularization and by producing multiple metalloproteases and other extracellular proteases that degrade different types of extracellular matrix molecules [24-28]. In this study, a highly pure population of EPI-NCSCs were obtained by virtue of their migratory ability through a minimally invasive procedure from the bulge of hair follicles. Isolated cells expressed both neural crest marker SOX10 and stem cell marker Nestin abundantly, which verifies their origin and multipotency.

Besides an appropriate cell type, the practicality of different routes of administration is another prominent factor in cell-based therapy. Cerebrospinal fluid, beyond its important role in the maintenance of extracellular ionic balance and providing a fluid cushion for the CNS, was recently implicated in carrying secreted proteins widely throughout life. Lately several studies have demonstrated the crucial role of CSF in neurogenesis at the brain-cerebrospinal fluid interface, regarding its various signaling factors [17,20]. Therefore it is not considered just as a watery fluid that bathes the brain and spinal cord. Moreover, CSF, owing to its circulatory system, which is in close contact with different parts of the CNS, provides a practical way for EPI-NCSCs transplantation. Above all, CSF-constituent proteins can play an instructive role in fate determination of EPI-NCSCs, as longSAGE gene-expression profile of these cells earlier revealed, EPI-NCSCs express some relevant growth-factor receptors that can convey CSF signals inside the cells [7].

Our data from culturing EPI-NCSCs in CSF has indicated that these cells can survive in this environment, and the expression of their pertinent markers proceeds for at least 72 hours, adequate for delivering cells to different parts of brain and spinal cord under *in vivo* conditions. Interestingly, EPI-NCSCs express early lineage markers like β-tubulin ІІІ and GFAP, which demonstrate that these cells can differentiate into either a neuronal or a glial lineage. Moreover, our investigation disclosed that CSF provides a trophic environment for proliferation of isolated EPI-NCSCs. However, the proliferation rate of these cells in CSF was significantly lower than that of cells in primary explants and expansion medium. This acquired trait of EPI-NCSCs after their cultivation in CSF is an attractive feature in cell-based therapy, because tumorigenicity of stem cells is one of the main setbacks of this approach.

Furthermore, our data show that CSF not only decreases the proliferation of EPI-NCSCs but also does not promote their differentiation toward any specific destiny, because the expression of early lineage genes in this medium diminished comparison with the primary explant. This condition can be appropriate for transplanted cells because it allows cells to differentiate according to instructive signals of their prospective target site.

It is noteworthy that the behavior of EPI-NCSCs in the current investigation was studied after their cultivation in healthy CSF, and this result may vary in different pathologic conditions. However, previously, Bai and his colleagues [29,30] showed that the injection of neural stem cells through the CSF is a practical method to graft cells into traumatic and diseased lesions of the spinal cord. Consistently, Satake *et al.* in 2004 [31] reported that transplanted mesenchymal stem cells can survive after a lumbar CSF injection and migrate into a previously created thoracic spinal cord injury.

## Conclusions

CSF, as a cocktail of growth factors, helps EPI-NCSCs to acquire some desirable traits. CSF, due to its circulatory system in close contact with different parts of the CNS, can be a practical route of administration for delivery of injected stem cells into various parts of the CNS. Further experiments are required to determine the fate, destiny, and behavior of EPI-NCSCs after their administration through CSF in spinal cord-injured animals or other models of neurodegenerative diseases.

## Abbreviations

BrdU: 5-Bromo-2′-deoxyuridine; cDNA: Complementary deoxynucleic acid; CEE: Chick embryo extract; CM: Cisterna magna; CNS: Central nervous system; CSF: Cerebrospinal fluid; Ct: Threshold cycle; ELISA: Enzyme-linked immunosorbent assay; EPI-NCSC: Epidermal neural crest stem cell; FBS: Fetal bovine serum; FGF: Fibroblast growth factor; HBSS: Hank balanced salt solution; HPRT: Hypoxanthine guanine phosphoribosyl transferase; IgG: Immunoglobulin G; PBS: Phosphate buffered saline; PI: Propidium iodide; qRT-PCR: Quantitative real-time polymerase chain reaction; RNA: Ribonucleic acid; RT: Room temperature; RT-PCR: Reverse transcriptase polymerase chain reaction; α-MEM: α-modified Eagle medium.

## Competing interests

The authors declare that they have no competing interests.

## Authors’ contributions

The first two authors contributed equally to the design of the study. They carried out the cellular and molecular experiments, analysis and interpretation of data, and manuscript writing. FA and AZ performed CSF collection and data analysis. MP participated in the design and coordination of the study and final approval of manuscript. All authors read and approved the final manuscript for publication.

## References

[B1] Sieber-Blum M Grim M Hu YF Szeder V Pluripotent neural crest stem cells in the adult hair follicle Dev Dyn 2004 231 258 269 10.1002/dvdy.20129 15366003

[B2] Mignone JL Roig-Lopez JL Fedtsova N Schones DE Manganas LN Maletic-Savatic M Keyes WM Mills AA Gleiberman A Enikolopov G Neural potential of a stem cell population in the hair follicle Cell Cycle 2007 6 2161 2170 10.4161/cc.6.17.4593 17873521 PMC3789384

[B3] Krejci E Grim M Isolation and characterization of neural crest stem cells from adult human hair follicles Folia Biol (Praha) 2010 56 149 157 20974047 10.14712/fb2010056040149

[B4] Sieber-Blum M Grim M The adult hair follicle: cradle for pluripotent neural crest stem cells Birth Defects Res C Embryol Today 2004 72 162 172 10.1002/bdrc.20008 15269890

[B5] Amoh Y Li L Katsuoka K Penman S Hoffman RM Multipotent nestin-positive, keratin-negative hair-follicle bulge stem cells can form neurons Proc Natl Acad Sci USA 2005 102 5530 5534 10.1073/pnas.0501263102 15802470 PMC556262

[B6] Clewes O Narytnyk A Gillinder KR Loughney AD Murdoch AP Sieber-Blum M **Human epidermal neural crest stem cells (hEPI-NCSC): characterization and directed differentiation into osteocytes and melanocytes** Stem Cell Rev 2011 7 799 814 10.1007/s12015-011-9255-5 21455606 PMC3252033

[B7] Hu YF Zhang ZJ Sieber-Blum M An epidermal neural crest stem cell (EPI-NCSC) molecular signature Stem Cells 2006 24 2692 2702 10.1634/stemcells.2006-0233 16931771

[B8] Sieber-Blum M Hu Y Epidermal neural crest stem cells (EPI-NCSC) and pluripotency Stem Cell Rev 2008 4 256 260 10.1007/s12015-008-9042-0 18712509

[B9] Morris RJ Liu Y Marles L Yang Z Trempus C Li S Lin JS Sawicki JA Cotsarelis G Capturing and profiling adult hair follicle stem cells Nat Biotechnol 2004 22 411 417 10.1038/nbt950 15024388

[B10] Sieber-Blum M Zhang JM Growth factor action in neural crest cell diversification J Anat 1997 191 493 499 10.1046/j.1469-7580.1997.19140493.x 9449068 PMC1467716

[B11] Garcez RC Teixeira BL Schmitt Sdos S Alvarez-Silva M Trentin AG Epidermal growth factor (EGF) promotes the in vitro differentiation of neural crest cells to neurons and melanocytes Cell Mol Neurobiol 2009 29 1087 1091 10.1007/s10571-009-9406-2 19415484 PMC11505785

[B12] Xia YX Ikeda T Xia XY Ikenoue T Differential neurotrophin levels in cerebrospinal fluid and their changes during development in newborn rat Neurosci Lett 2000 280 220 222 10.1016/S0304-3940(00)00782-5 10675800

[B13] Miyan JA Zendah M Mashayekhi F Owen-Lynch PJ Cerebrospinal fluid supports viability and proliferation of cortical cells in vitro, mirroring in vivo development Cerebrospinal Fluid Res 2006 3 2 10.1186/1743-8454-3-2 16549001 PMC1450312

[B14] Martin C Bueno D Alonso MI Moro JA Callejo S Parada C Martin P Carnicero E Gato A FGF2 plays a key role in embryonic cerebrospinal fluid trophic properties over chick embryo neuroepithelial stem cells Dev Biol 2006 297 402 416 10.1016/j.ydbio.2006.05.010 16916506

[B15] Zappaterra MD Lisgo SN Lindsay S Gygi SP Walsh CA Ballif BA A comparative proteomic analysis of human and rat embryonic cerebrospinal fluid J Proteome Res 2007 6 3537 3548 10.1021/pr070247w 17696520

[B16] Salehi Z Mashayekhi F Naji M Pandamooz S Insulin-like growth factor-1 and insulin-like growth factor binding proteins in cerebrospinal fluid during the development of mouse embryos J Clin Neurosci 2009 16 950 953 10.1016/j.jocn.2008.09.018 19359179

[B17] Lehtinen MK Zappaterra MW Chen X Yang YJ Hill AD Lun M Maynard T Gonzalez D Kim S Ye P D’Ercole AJ Wong ET LaMantina AS Walsh CA The cerebrospinal fluid provides a proliferative niche for neural progenitor cells Neuron 2011 69 893 905 10.1016/j.neuron.2011.01.023 21382550 PMC3085909

[B18] Sawamoto K Wichterle H Gonzalez-Perez O Cholfin JA Yamada M Spassky N Murcia NS Garcia-Verdugo JM Marin O Rubenstein JL Tessier-Lavigne M Okano H Alvarez-Buylla A New neurons follow the flow of cerebrospinal fluid in the adult brain Science 2006 311 629 632 10.1126/science.1119133 16410488

[B19] Kim S Lehtinen MK Sessa A Zappaterra MW Cho SH Gonzalez D Boggan B Austin CA Wijnholds J Gambello MJ Malicki J LaMantia AS Broccoli V Walsh CA The apical complex couples cell fate and cell survival to cerebral cortical development Neuron 2010 66 69 84 10.1016/j.neuron.2010.03.019 20399730 PMC2872122

[B20] Lehtinen MK Walsh CA Neurogenesis at the brain-cerebrospinal fluid interface Annu Rev Cell Dev Biol 2011 27 653 679 10.1146/annurev-cellbio-092910-154026 21801012 PMC3777264

[B21] Jackson JS Golding JP Chapon C Jones WA Bhakoo KK Homing of stem cells to sites of inflammatory brain injury after intracerebral and intravenous administration: a longitudinal imaging study Stem Cell Res Ther 2010 1 17 10.1186/scrt17 20550687 PMC2905093

[B22] Sieber-Blum M Schnell L Grim M Hu YF Schneider R Schwab ME Characterization of epidermal neural crest stem cell (EPI-NCSC) grafts in the lesioned spinal cord Mol Cell Neurosci 2006 32 67 81 10.1016/j.mcn.2006.02.003 16626970

[B23] Sieber-Blum M Epidermal neural crest stem cells and their use in mouse models of spinal cord injury Brain Res Bull 2010 83 189 193 10.1016/j.brainresbull.2010.07.002 20637266

[B24] Amoh Y Li L Katsuoka K Hoffman RM Multipotent hair follicle stem cells promote repair of spinal cord injury and recovery of walking function Cell Cycle 2008 7 1865 1869 10.4161/cc.7.12.6056 18583926

[B25] Hu YF Gourab K Wells C Clewes O Schmit BD Sieber-Blum M Epidermal neural crest stem cell (EPI-NCSC)–mediated recovery of sensory function in a mouse model of spinal cord injury Stem Cell Rev 2010 6 186 198 10.1007/s12015-010-9152-3 20414748 PMC2887506

[B26] Amoh Y Li L Campillo R Kawahara K Katsuoka K Penman S Hoffman RM Implanted hair follicle stem cells form Schwann cells that support repair of severed peripheral nerves Proc Natl Acad Sci USA 2005 102 17734 17738 10.1073/pnas.0508440102 16314569 PMC1308908

[B27] Amoh Y Aki R Hamada Y Niiyama S Eshima K Kawahara K Sato Y Tani Y Hoffman RM Katsuoka K Nestin-positive hair follicle pluripotent stem cells can promote regeneration of impinged peripheral nerve injury J Dermatol 2012 39 33 38 10.1111/j.1346-8138.2011.01413.x 22098554

[B28] Liu F Uchugonova A Kimura H Zhang C Zhao M Zhang L Koenig K Duong J Aki R Saito N Mii S Amoh Y Katsuoka K Hoffman RM The bulge area is the major hair follicle source of nestin-expressing pluripotent stem cells which can repair the spinal cord compared to the dermal papilla Cell Cycle 2011 10 830 839 10.4161/cc.10.5.14969 21330787

[B29] Wu S Suzuki Y Kitada M Kataoka K Kitaura M Chou H Nishimura Y Ide C New method for transplantation of neurosphere cells into injured spinal cord through cerebrospinal fluid in rat Neurosci Lett 2002 318 81 84 10.1016/S0304-3940(01)02488-0 11796191

[B30] Bai H Suzuki Y Noda T Wu S Kataoka K Kitada M Ohta M Chou H Ide C Dissemination and proliferation of neural stem cells on the spinal cord by injection into the fourth ventricle of the rat: a method for cell transplantation J Neurosci Methods 2003 124 181 187 10.1016/S0165-0270(03)00007-4 12706848

[B31] Satake K Lou J Lenke LG Migration of mesenchymal stem cells through cerebrospinal fluid into injured spinal cord tissue Spine (Phila 1976) 2004 29 1971 1979 10.1097/01.brs.0000138273.02820.0a 15371697

